# A Five-Year Trend of Intestinal Parasite Prevalence among Students Attending Clinic at University of Gondar, Northwest Ethiopia

**DOI:** 10.1155/2021/8897935

**Published:** 2021-02-20

**Authors:** Adane Derso, Gizachew Yenealem, Ayenew Addisu

**Affiliations:** ^1^Department of Medical Parasitology, School of Biomedical and Laboratory Sciences, College of Medicine and Health Sciences, University of Gondar, Gondar, Ethiopia; ^2^School of Biomedical and Laboratory Sciences, College of Medicine and Health Sciences, University of Gondar, Gondar, Ethiopia

## Abstract

**Background:**

Intestinal parasitic infections are the cause of the highest worldwide infectious disease and the major public health problems in developing countries. Among the cases, children and younger age are at high risk and the major victims. The aim of this study was to assess the five-year trend of intestinal parasite prevalence among University of Gondar students.

**Method:**

A retrospective cross-sectional study was conducted to assess the trend of intestinal parasite prevalence among students at the University of Gondar. The data was collected from students who have studied at the University of Gondar from 2014 to 2018 and who visited the student's clinic and had recorded results of stool sample diagnosis on the laboratory logbook. Stool specimens were examined using direct saline wet mount methods. The data was analyzed by using SPSS version 20 software, and *P* value < 0.05 was considered as statistically significant. Moreover, chi-square was used to assess the association of different variables.

**Result:**

During the study period, a total of 6244 stool samples were requested for intestinal parasite diagnosis and it was found that 2850 specimens were positive for intestinal parasites, representing an overall prevalence of 45.6% with a fluctuating trend. Ten different parasites were reported with *Entamoeba histolytica/dispar* (20.3%) and *Giardia lamblia* (8.2%), the most frequently detected intestinal parasites. The prevalence of intestinal parasitic infections was higher in males (35.4%) than females (10.2%) (*P* = 0.02).

**Conclusions:**

Intestinal parasitic infection was highly prevalent, and there were fluctuations in the prevalence of intestinal parasites from 2014 to 2018. Environmental sanitation improvement and health education schemes at the University of Gondar can be considered quite indispensable for the prevention and control of parasitic infections in the area.

## 1. Background

Intestinal parasites are among protozoan and helminth groups that have been known to compromise the quality of human life since prehistoric times. These parasites cause nutrient deficiencies, anaemia, and impaired growth and cognitive development on human health [1, 2]. Intestinal parasitic infections, which are more common in developing countries, are widely distributed throughout the world. These infectious parasites can be transmitted through skin penetration, ingestion of water, and soil or food contaminated by faeces containing the infective stage of the pathogen. Commonly, clinical features of the parasites on humans were asymptomatic but act as carriers and as sources of infection within their community [2, 3].

Even though the focus of disease-combating efforts has moved forward, the disease incidence data indicates that intestinal parasitic diseases continue to weaken human productivity across the globe [2], The morbidity and mortality of parasites with or without other disease are the most common occasion which affected 820 million populations with roundworms and others with pathogenic intestinal protozoan parasites 17.46% and 0.87% for *Giardia lamblia* and *Entamoeba histolytica*/*dispar*, respectively [4, 5].

The intestinal parasitic infection main contributors include public and individual hygienic activities, community awareness about the prevention and control mechanisms of parasitic infections, access to the safe food and water sources, economic and educational status of the population, previous control efforts, and availability of toilet [6–8].

In this regard, University students who live together in campuses and share different materials for their day to day activities may expose them to intestinal parasitic infections. Therefore, this study is important to know the five-year trend of intestinal parasitic infection among students at the University of Gondar. In higher institutions, like the University of Gondar, there is insufficient information on the magnitude of parasitic infections. The information generated from this study may provide the initial information needed for planning control measures. Besides, identification of parasitic agents is an important step for the initiation of deliberate treatment strategies.

## 2. Methods

### 2.1. Study Area and Period

An institutional-based retrospective cross-sectional study was employed to assess the trend prevalence of intestinal parasite infection among students at the University of Gondar, Gondar, Northwest Ethiopia. The study was conducted in students' clinic located at different campuses of the university, namely, College of Medicine and Health Sciences, College of Social Science and Humanities, Natural and Computational Science, College of Veterinary Medicine and Animal Science, College of Business and Economics, and College of Agriculture and Rural Transformation. Within each campus, there is students' clinic providing clinical diagnosis and treatment activities. The total professional workers in the clinic included 30 nurses, 15 laboratory technologists, and 15 pharmacy professionals. The study was carried out from 2014 to 2018.

### 2.2. Study Design

An institutional-based retrospective cross-sectional study was used to assess the trend of intestinal parasite prevalence among students at the University of Gondar, Northwest Ethiopia, 2019.

### 2.3. Study Participants and Data Collection

The study participants were all students who have studied at the University of Gondar from the years 2014–2018, visited students' clinic, and had recorded results of stool sample diagnosis on laboratory logbook. Students whose names and sociodemographic characteristics were not well documented in the registration logbook were excluded from the study even if they provided stool samples. Sociodemographic and laboratory results were reviewed from the laboratory registration logbook using a checklist. All laboratories in the clinics have been providing results of stool examination through the wet mount technique. After stool examinations were completed, infected students were treated based on the national guideline.

### 2.4. Data Analysis and Interpretation

After the data was entered, cleaned, and checked, it was analyzed using SPSS version 20 software, chi-squares were used to test the association of variables, and the *P* value less than 0.05% was taken as statistically significant.

## 3. Result

### 3.1. Sociodemographic Characteristics and Prevalence of Intestinal Parasitic Infections at University of Gondar Students' Clinic

A total of 6244 stool samples were examined from 2014 to 2018 at the University of Gondar students' clinic. Of the examined stool sample, about 1482 (23.7%) and 4762 (76.3%) were from female and male students, respectively. The ages of study participants ranged from 18 to 35 years. The mean age of the study participants was 21 years.

In 2018, 1779 stool samples were requested for intestinal parasite diagnosis, which was the highest (28.5%), while the least number of intestinal parasite diagnosis was found in 2014 (9.4%) (Table 1). During the study period, from a total of 6244 stool samples examined and registered on the lab logbook, 2850 were positive for intestinal parasites which accounts for 45.6% of the study participants.

Throughout the years, the records for males were more than the female counterparts. Furthermore, the prevalence of intestinal parasitic infections was higher in males 35.4% than females 10.2%; this difference is statistically significant (2213/2850 (35.4%) vs. 637/2850 (10.2%) (*P* = 0.02), respectively). Thus, being a male or female had influence on the risk of infection.

**Figure 1:**
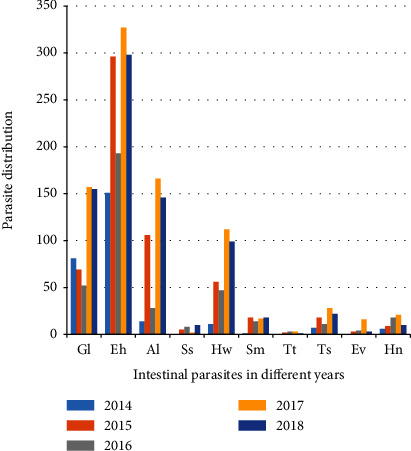
Intestinal parasite distribution at University of Gondar students' clinic, Gondar, Northwest, Ethiopia. Gl: Giardia lamblia; Eh: Entamoeba histolyitca/dispar; Al: Ascaris lumbricoides; Ss: Strongyloides stercoralis; Hw: hookworm; Sm: Schistosoma mansoni; Tt: Trichuris trichiura; Ts: Taenia species; Ev: Enterobius vermicularis; Hn: Hymenolepis nana.

Two protozoan species and eight helminth species (Figure 1) of the intestinal parasites were found. *Entamoeba histolytica/dispar* was the most predominant parasite (20.3%) followed by *G. lamblia* (8.2%). Of the helminths identified, *Ascaris lumbricoides* (7.4%) is the most prevalent helminthic parasite followed by hookworm, *Taenia* species, *Hymenolepis nana, Schistosoma mansoni*, *Strongyloides stercoralis*, *Enterobius vermicularis*, and *Trichuris trichiura* with the prevalence of 5.2%, 1.4%, 1.2%, 1.1%, 0.4%, 0.4%, and 0.1%, respectively. One hundred eighty-five and eight participants were found with double and triple intestinal parasite infection, respectively.

### 3.2. Annual Trends of the Prevalence of Intestinal Parasites at University of Gondar Students' Clinic

**Figure 2:**
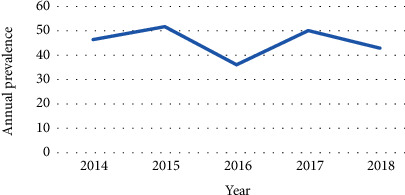
Trend of intestinal parasite prevalence among students at University of Gondar student's clinic, 2014–2018.

The results of this study indicate fluctuating trends of intestinal parasites in 5 years of the study period, with the highest prevalence of intestinal parasite reported in 2015 with the prevalence of 584/1130 (51.7%) and the lowest, 379/1050 (36.1%), in 2016 (Figure 2).

## 4. Discussion

Maintaining regular surveillance and observing the trend distribution of intestinal parasitic infections in a given community is a prerequisite for planning and evaluation of the existing and aids to formulate appropriate intervention program. In line with this, the present study attempted to retrospectively analyze the trend of common intestinal parasites in a 5-year period at the University of Gondar students' clinic, Gondar, Northwest Ethiopia.

This study finding showed a cyclic trend pattern of intestinal parasite prevalence in the last five years. The finding is important to formulate prevention and control strategies, for budget allocation, and resource mobilization and also to empower the laboratories for the coming years at the University of Gondar.

The overall prevalence of intestinal parasite infection in this study was 45.6%, which agreed with a study in Gondar, Poly Health Center (41.3%) [9]. But it was higher than other studies done in different parts of Ethiopia: Bale-Robe (6.23%), Mojo Health Center (9.3%) [10, 11], and other countries' reports [12–14]. These differences in prevalence could be due to the use of different diagnostic methods and sociodemographic differences of the study subjects and geographic condition of the study area.

In the present retrospective study, ten different types of intestinal parasites were found in the stool examination. Protozoan infections (28.5%) were more common than helminth infections (17.2%). Like in the present study, protozoans were more prevalent in studies from Poly Health Center (68.2) [9], Bale-Robe Health Center (47.1%) [10], and Wonago Health Center (78.3%) [15]. This higher prevalence of these two protozoan parasites agreed with the report of the WHO which showed these two parasites as common causes of intestinal infection throughout Ethiopia [16]. Similarly, studies from Ho Teaching Hospital, Ghana [12], Gaza Strip, Northern India (90.5%), and White Nile State, Sudan (54.1%), showed protozoans were more prevalent than helminths [17–19]. However, the present study was inconsistent with the study conducted in Saudi Arabia [13] which revealed soil-transmitted helminth as the most predominant intestinal parasite. The higher prevalence of protozoan parasites may be associated with poor personal and environmental hygiene since the mode of transmission of these parasites was mainly through the feco-oral route.

*Entamoeba histolytica/dispar* was the predominant parasite identified with a prevalence of 20.3%, lower than the previous study in Wonago Health Center, Southern Ethiopia (53.8%) [15], however, higher than studies conducted in Poly Health Center, Gondar (16.8%) [9], and Hawassa University students' clinic, Southern Ethiopia (18%) [20], and other countries' studies: in South India (8.2%) [21], rural Haryana (5.4%) [22], Ho Teaching Hospital, Ghana (5.7%) [12], and Guatemala (16.1%) [23]. Besides this, the finding of this study showed significantly higher than studies done in Saudi Arabia (0.27%) [13], Istanbul, Turkey (0.05%) [24]. These differences might be due to diagnostic methods employed like the absence of molecular-based diagnosis to differentiate *E. histolytica* from *E. dispar* at the University of Gondar students' clinic. In addition, it might be due to low sanitary condition, contamination of drinking water or vegetables.

This study indicated that variation in the prevalence of intestinal parasite showed fluctuating overall trend of intestinal parasitic infections in the study area. A decreasing number of infections occurred in 2014 compared to 2015. However, there was an increment in the number of cases from 2016 to 2017, followed by a slight decline in the number of cases in 2018. Similar studies were found in Mojo town [11], Gondar at Poly Health Center [9], and India [19] that reported a fluctuating trend of intestinal parasite infection. However, the finding of this study differed from a study conducted in Ethiopia [10] which revealed an increasing and in Morocco [25] a decreasing trend of parasitic infection. The possible trend variation might be due to the difference in the prevention and control strategies in place by different countries/institutions, geographical differences, and socioeconomic conditions of the study subjects.

The present study showed a higher rate of infection in males than females, 35.4% and 10.2%, respectively. However, our finding differed from studies in Mojo Health Center, Hawassa University students' clinic, Wonago Health Center in Ethiopia [11, 15, 20], and Bugando Medical Center in Tanzania [26] which reported females as the more infected group. The differences in the proportion of parasitic infection between males and females might be due to genetic and behavioral differences and hormonal and immunological operation mechanisms such as the immunomodulatory effects of testosterone in males which increases their susceptibility to certain parasitic infections [27].

## 5. Conclusion

Intestinal parasitic infection was highly prevalent (45.6%), and the finding showed cyclic pattern fluctuations in the trend prevalence of intestinal parasite infection. The proportions of infection were higher in protozoan compared to helminths. It is necessary to develop effective prevention and control strategies including health education to the university community and improvement of personal hygiene and environmental sanitation. Besides, a scheduled checkup for intestinal parasite diagnosis to food handlers (cafe workers) will be advisable.

## 6. Limitation of the Study

Due to the study type, retrospective study, we were unable to get behavioral and environmental data which could have been predisposing factors for intestinal parasitic infection. Since the laboratory method employed for the identification of intestinal parasites at students' clinic was mainly saline wet mount preparation, we are unable to differentiate *E. histolytica* and *E. dispar* due to their morphological similarities.

## Figures and Tables

**Table 1:** Prevalence of intestinal parasites across years among students attending University of Gondar students' clinic, 2014–2018.

Year	Total tested samples No. (%)	Proportion of positive samples No. (%)
2014	588 (9.4)	273 (46.4)
2015	1130 (18.1)	584 (51.7)
2016	1050 (16.8)	379 (36.1)
2017	1697 (27.2)	850 (50.1)
2018	1779 (28.5)	764 (42.9)

## Data Availability

All data generated or analyzed during this study are included in this published article.

## Abbreviations

Gl: *Giardia lamblia*
Eh: *Entamoeba histolytica/dispar*
Al: *Ascaris lumbricoides*
Ss: *Strongyloides stercoralis*
Hw: Hookworm
Sm: *Schistosoma mansoni*
Tt: *Trichuris trichiura*
Ts: *Taenia species*
Ev: *Enterobius vermicularis*
Hn: *Hymenolepis nana.*
